# Quality Assessment of Established and Emerging Blood Components for Transfusion

**DOI:** 10.1155/2016/4860284

**Published:** 2016-12-14

**Authors:** Jason P. Acker, Denese C. Marks, William P. Sheffield

**Affiliations:** ^1^Centre for Innovation, Canadian Blood Services, Edmonton, AB, Canada; ^2^Department of Laboratory Medicine and Pathology, University of Alberta, Edmonton, AB, Canada; ^3^Research and Development, Australian Red Cross Blood Service, Sydney, NSW, Australia; ^4^Centre for Innovation, Canadian Blood Services, Hamilton, ON, Canada; ^5^Department of Pathology and Molecular Medicine, McMaster University, Hamilton, ON, Canada

## Abstract

Blood is donated either as whole blood, with subsequent component processing, or through the use of apheresis devices that extract one or more components and return the rest of the donation to the donor. Blood component therapy supplanted whole blood transfusion in industrialized countries in the middle of the twentieth century and remains the standard of care for the majority of patients receiving a transfusion. Traditionally, blood has been processed into three main blood products: red blood cell concentrates; platelet concentrates; and transfusable plasma. Ensuring that these products are of high quality and that they deliver their intended benefits to patients throughout their shelf-life is a complex task. Further complexity has been added with the development of products stored under nonstandard conditions or subjected to additional manufacturing steps (e.g., cryopreserved platelets, irradiated red cells, and lyophilized plasma). Here we review established and emerging methodologies for assessing blood product quality and address controversies and uncertainties in this thriving and active field of investigation.

## 1. Introduction

**Figure 1:**
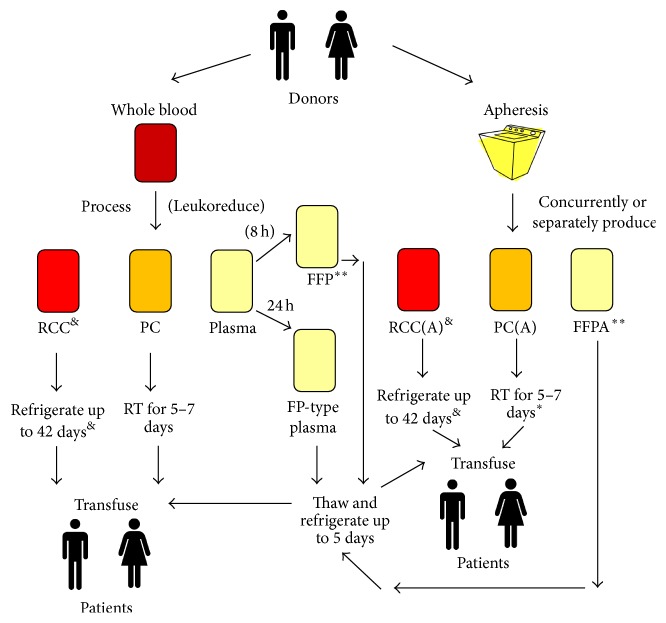
Schematic diagram of blood component manufacturing. Donations are either whole blood (left branch) or apheresis (right branch). At left, whole blood donations are processed into red cell concentrates (RCCs), platelet concentrates (PCs), or (transfusable) plasma, with or without leukoreduction by filtration. At right, apheresis donations (A) yield RCC(A), PC(A), or FFPA; some products may be made concurrently (e.g., FFPA and PC(A)). FFP is frozen within 8 hours in some jurisdictions or may be defined by quality control standards in others. FP-type plasma is frozen within 24 hours of phlebotomy. FFP or FFPA may be thawed and stored refrigerated up to 5 days prior to transfusion in some jurisdictions, while RCC or RCC(A) may be refrigerated no more than 42 days and platelets are typically stored at RT for 5–7 days, although ^*∗*^the FDA allows refrigeration and transfusion of cold-stored platelets for 72 hours; cryopreserved platelets are also under investigation. ^*∗∗*^FFP or FFPA may be further manipulated by drying or pathogen reduction treatment. ^&^RCC may be further manipulated, for example, by washing, irradiation, or cryopreservation, in licensed procedures that may reduce shelf-life.

Blood component therapy became the standard of care in transfusion medicine throughout the industrialized world in the latter half of the twentieth century. The widespread adoption and retention of component therapy were driven by innovations in refrigeration, blood bag design, anticoagulant and preservative solution composition, infectious disease testing, and other means of donor screening [1]. The traditional trio of blood components are red cell and platelet concentrates and plasma, which may be generated either by the processing of whole blood donations or via apheresis. Whole blood is processed by centrifugation, predominantly by one of two main protocols which generate different intermediates: platelet-rich plasma (PRP) or a buffy coat (BC) [2]. White blood cells may be removed from blood components through the use of leukoreduction filters, often during blood processing and before storage [3]. Blood components require different storage conditions, with plasma being frozen, red cells being refrigerated, and platelets being maintained at ambient room temperature (RT) (see Figure 1 for an overall schematic diagram of component manufacturing). Blood component therapy remains widely practiced and widely supported for the majority of patients requiring transfusions; however in the trauma setting it has been suggested that whole blood may be superior to component therapy [4]. Although out of the scope of this review, this controversial concept is under active investigation. This article reviews issues, concepts, methodology, and challenges in assessing the quality of blood components and is not limited only to the traditional trio but also addresses emerging products such as cryopreserved platelets and lyophilized plasma. Below we explore each component in this context in detail, in no particular order.

## 2. Overview: Assessing the Quality of Transfusable Plasma

Plasma is the liquid portion of an anticoagulated blood donation. Ideally, transfusable plasma would be manufactured under controlled conditions and assayed, prior to its release, for in vitro activities known to correlate with efficacy in each of its clinical indications. The first aspect has been consistently achieved, with impeccable control readily demonstrated by manufacturers. However, with respect to the second aspect, that of quality testing, there is currently no single test or combination of tests established to correlate with clinical efficacy of transfusable plasma. Moreover, quality testing is typically done on a portion of units selected from inventory during the product's shelf-life; prerelease testing is currently done only for transmissible disease screening [5]. Linking quality markers to outcomes of plasma transfusion has not been achieved to date, in large part due to the weakness of clinical evidence of efficacy for most indications for which plasma is transfused. Physicians nevertheless transfuse plasma frequently, relying for guidance on clinical experience and expert opinion. Manufacturers of transfusable plasma and some regulatory bodies therefore rely upon surrogate measures assumed to correlate with therapeutic efficacy. These measures typically relate to one or more labile coagulation factor whose decline might be an early indicator of decreased quality. Investigational quality assessment has evolved beyond the simple determination of regulated coagulation factor activities, towards the assessment of as many coagulation-related protein activities as is feasible, and some work has also been done on global assays of coagulation such as thrombin generation and viscoelastic testing.

### 2.1. Indications and Rationale for Plasma Transfusion

Most recommendations from national or professional bodies indicate therapeutic plasma transfusion for the correction of clotting factor deficiencies in patients who are bleeding or prophylactic transfusion for those judged to be at risk of bleeding [6–12]. The deficiencies may be of single clotting factors for which no appropriate concentrate is available to the treating physician, or of two or more clotting factors, in the setting of disseminated intravascular coagulation (DIC), vitamin K antagonist reversal, liver disease, cardiopulmonary bypass (CPB), or massive transfusion. Plasma transfusion, or more specifically plasma exchange (PEX), is also indicated in the treatment of thrombotic thrombocytopenic purpura (TTP).

Most indications for plasma transfusion are tied to coagulopathy, defined by Hunt as “a condition in which the blood's ability to clot is impaired” [13]. Providing plasma by transfusion to remedy coagulopathy is biologically plausible, given that plasma contains all of the soluble coagulation factors, with the caveat that platelets and red cells also contribute to hemostasis, the balanced state in which blood loss from injury is quickly stemmed by clotting [14]. For plasma proteins for which no purified concentrate is available (e.g., Factor V or Protein S), transfusable plasma is the only replacement source available to the clinician treating such hereditary or acquired single factor deficiencies. In DIC and CPB, coagulation factor consumption and/or bypass pump fluid management procedures reduce circulating levels of multiple coagulation factors. Vitamin K antagonists such as warfarin reduce the plasma concentration of functional factors (F) II, VII, IX, and X (FII, FVII, FIX, FX) and Proteins C and S, all of which can be found in greater concentration in donor plasma than that of the warfarinized patient, if the pharmacotherapy needs to be rapidly reversed. As the liver is the site of synthesis of most coagulation factors and most coagulation inhibitors, plasma transfusion seems logical to assist the bleeding patient with liver disease, unless hemostasis has been rebalanced. In massive transfusion, usually defined based on the number of red cell units transfused, it is hard to envisage clinical care specialists being able to establish hemostasis without the provision of the coagulation factors found in plasma. Nevertheless, plasma is a relatively dilute source of many coagulation factors that can only be administered relatively slowly, and biological plausibility is no substitute for high-level clinical evidence to guide physician practice.

### 2.2. Evidence Base for Plasma Transfusion

High quality clinical evidence of plasma transfusion for most of its indications is sparse. In overlapping systemic reviews covering 1964–2011, Yang et al. [15] and Stanworth et al. [16] found 80 completed or near-completed randomized clinical trials (RCT) addressing the efficacy of this medical intervention. No picture of significant benefit across most clinical indications for plasma transfusion emerged, with most studies being judged to be underpowered. Murad et al. reported similar findings [17]. Prophylactic use of plasma transfusion in particular was found to be unsupported by evidence. There were sufficient trials in the area of cardiac surgery to perform a meta-analysis, but in this setting no benefit could be ascribed to plasma transfusion with respect to 24-hour postoperative blood loss [15].

Plasma exchange for TTP is arguably the indication for which the strongest evidence exists, specifically showing a survival benefit of plasma exchange (PEX) versus plasma infusion (PI) in two RCTs with appropriate randomization [18, 19]. Meta-analysis of these trials showed a significant reduction of mortality after seven PEX procedures, to about 31% of that seen with PI [20]. PEX may be effective in TTP due to removal of anti-ADAMTS13 autoantibodies, removal of pathological ultra-large von Willebrand factor (VWF) multimers, and/or via provision of transfused ADAMTS13.

In critically bleeding patients requiring massive transfusion, considerable retrospective data led many military and civilian trauma specialists to adopt formula-driven treatment protocols, in a ratio of one red cell unit (and platelet unit) to one plasma unit, over the last decade or so [21]. An RCT has now tested this approach: the Pragmatic Randomized Optimal Platelet and Plasma Ratios (PROPPR) trial [22]. This RCT randomized 680 patients arriving at Level 1 trauma centres in North America directly from the scene of their injury, and predicted to require massive transfusion, to either a 1 : 1 : 1 plasma : platelets : red cell unit treatment ratio or a 1 : 1 : 2 ratio. No difference in 24-hour or 30-day mortality, the primary outcomes, was found, although, in the ancillary outcome of cause of death in the first 24 hours, death by exsanguination was significantly reduced in the 1 : 1 : 1 group. The results may provide some support for a plasma-rich treatment approach.

Blood component therapy, including plasma transfusion, was introduced into medical practice prior to the establishment of modern evidence-based medicine. Most RCTs (excepting those in TTP) have failed to find evidence of benefit for plasma transfusion. It is not known whether this failure derives from a true lack of benefit or from limitations in trial design for those studies undertaken to date. This uncertainty and the sparseness of evidence complicate the task of assessing the quality of transfusable plasma as a biological product but have not discouraged the evolution of an extensive literature on plasma quality.

### 2.3. Nature and Amounts of Coagulation-Related Proteins in Plasma

Proteomics has demonstrated that human plasma contains thousands of different proteins, present over a range of concentration spanning 10–12 orders of magnitude [23, 24]. Coagulation-related proteins include fibrinogen and coagulation factors II (prothrombin), V, VII, VIII, X, XI, XII, and XIII, as well as von Willebrand Factor (VWF). ADAMTS13 is also of special interest due to its role in TTP. Major coagulation inhibitors include Proteins C and S, antithrombin, alpha-1-antitrypsin, and C1-esterase inhibitor. Among the fibrinolytic proteins, plasminogen and alpha-2-antiplasmin are the most abundant. Most of these proteins demonstrate a wide normal range in healthy blood donors. Reference ranges for coagulation factor activity levels (defined as the 95% confidence limit of the mean ± two SD) were found to vary between the smallest interval of 0.65–1.3 IU/mL for prothrombin and the largest interval of 0.40–1.9 IU/mL for FXII, in a study of over 400 normal men and women [25].

Which factors patients require, and how much of these factors need to be present in transfusable plasma, is unclear, because different plasma proteins appear to be present in excess of physiological requirements, with different reserve margins. FVIII levels between 0.05 and 0.40 IU/mL are considered to result in only a mild bleeding tendency. In contrast, fibrinogen levels only slightly below the lower limit of the normal range have been suggested as triggers for fibrinogen supplementation in bleeding patients [26]. Apart from FIX and FVIII, most coagulation-related protein genes are not sex-linked but are instead found on autosomes. In consequence, their deficiencies are recessive and require mutation or inactivation of both gene copies; 50% levels of most coagulation factors, therefore, fail to cause clinically relevant symptoms. Rare individuals with genetic deficiencies of FV, FVII, FX, or FXIII typically do not present with a bleeding diathesis unless their circulating levels of these proteins fall below 0.05 IU/mL [27]; in contrast some individuals with prothrombin deficiency exhibit a bleeding diathesis with activity levels as high as 18.9% [28].

A threshold of coagulation factor levels of 0.30 IU/mL (30% of normal) is often cited as being sufficient to support clinical or surgical hemostasis. At least one textbook asserts this concept without attribution. Evidence in support of this estimated threshold appears to come from two sources: a 1961 review of surgical experience in patients with single factor deficiencies [29] and surgical studies from the 1980s and 1990s in which shed blood was replaced with plasma-poor red cell concentrates [30]. Aggeler found coagulation factor levels of 20–30% of normal sufficient to avoid bleeding complications during surgery in single factor deficiency patients [29]. Murray et al. replaced blood losses occurring during major elective surgery with colloid solutions and packed washed red cells to maintain a hematocrit of 30% [30]. Replacement of one blood volume did not provoke bleeding from incision edges or bleeding judged to be atypical for a specific surgery, in a small series of 12 patients. Excessive bleeding, as clinically defined, was found after greater than one calculated blood volume replacement. Hiippala et al. performed a similar study in 60 patients undergoing major urological or abdominal surgery, finding by regression analysis that persistent oozing of incision edges or surgical fields occurred after 118 ± 17% blood volumes, when prothrombin levels were 34 ± 5% of normal, similar to the expected 70% pan-factor depletion calculated on the basis of exchange of one volume of blood with equivalent packed red cells [31]. Erber summarized these studies as defining a critical level of 30% clotting factor activity required to maintain hemostasis, also defining a fibrinogen concentration of 1.0 g/L and a platelet count of 100 × 10^9^/L as critical values [32].

The 30% threshold concept provides a potential explanation for the difficulty in demonstrating the clinical benefit of plasma transfusion in coagulopathy: the “zone of therapeutic anticoagulation” is relatively narrow [33]. Many patients feared to be at risk of bleeding on the basis of elevated laboratory clotting time values are not actually at risk of bleeding; and some patients with severely traumatic blood losses are not salvageable. In the plasma quality field, such uncertainties have given rise to competing perspectives. If the goal of plasma transfusion therapy is to restore clotting factor levels to 30%, then from first principles this goal can be achieved more rapidly, and with the infusion of lower volumes of plasma, if mean factor levels in the product are as high as possible (i.e., 100% versus lower levels). On the other hand, if some coagulation proteins are less important determinants of coagulability than others, then too great an attention to their preservation could be wasteful in terms of the efficiency of donated blood processing.

### 2.4. Labile Coagulation Factors

Some coagulation factors decline more rapidly during plasma processing or storage than others. In 1979, Counts et al. reported that FV and FVIII were the only clotting factors to decline to less than 50% of initial values in modified whole blood from which platelets had been removed, over 14 days of refrigerated storage [34]. In 1984, Kakaiya et al. reported that extending the refrigeration of whole blood from 6 to 18–20 hours prior to separation of plasma led to significant losses in FVIII, but not FV activity [35]. More recently, in a multicentre, Biomedical Excellence for Safer Transfusion (BEST) Collaborative study employing a pool-and-split design, whole blood was cooled to ambient temperature (18–25°C) and processed into plasma under two conditions: less than 8-hour hold or 24-hour hold. FVIII was the most affected factor, declining 23% on average relative to the shorter hold; FV, FVII, FXI, FXII, FXIII, VWF, and antithrombin were unaffected. FII, FIX, and FX declined <5%, while Proteins C and S lost 6 and 14% activity, respectively [36]. A stability study of thawed refrigerated plasma over 120 hours showed that FVIII and FV activity significantly declined, by 8.5% and 27% within 24 hours, but that fibrinogen remained stable and that FVII remained stable for 48–72 hours of refrigerated storage [37]. Overall, the results suggest that FVIII is the most consistently labile of the major coagulation-related proteins in plasma, with FV, Protein C, and Protein S showing some instability in some studies.

In spite of the reproducibility of FVIII instability, its molecular cause is not well understood. FVIII is a heterodimer stabilized in plasma by copper ions and via binding to VWF [38]. Loss of the copper ion leads to dissociation and loss of activity. Activation of FVIII to FVIIIa by controlled proteolysis by thrombin also promotes dissociation from VWF. FVIIIa loses activity spontaneously due to dissociation of its A2 domain or via proteolytic attack by Activated Protein C. It is not known whether storage in anticoagulated blood or plasma promotes loss of activity through either mechanism or whether the instability of FVIII is due to alternative causes.

### 2.5. Plasma versus Modified Plasma for Transfusion

Plasma for transfusion is obtained either by processing of anticoagulated whole blood or from apheresis systems. Most blood operators exploit the ability to freeze plasma to extend its shelf-life, although never-frozen liquid plasma is licensed in some locations (e.g., the USA) [39]. Thawed plasma may be stored in a refrigerated state for lengths of time varying from hours to days, depending on the jurisdiction and the specific details of how it was handled and frozen prior to thawing. Several modified versions of plasma also offer enhancements over the traditional products with respect to ease of administration or safety. Dehydrated plasma is stable at room temperature and can therefore be more easily transported into austere environments remote from hospitals, rapidly rehydrated, and administered in the field [40]. Specifically, lyophilized plasma is available for military and/or civilian use in some countries, and spray-dried plasma is under investigation. Pathogen-reduced plasma is also in routine clinical use in many countries; this product comprises plasma to which a nucleic acid-damaging additive that can be photochemically activated has been added. Quality comparisons of modified plasma to standard plasmas are readily available, but they suffer from the same difficulties in terms of extrapolation to predicting clinical efficacy of the standard plasmas, due to the unsatisfactory evidence base [16].

### 2.6. “Standard” Forms of Transfusable Plasma and Regulations: FFP and FP-Type Plasma

Transfusable plasma units vary with respect to the time from phlebotomy to the frozen state and the methods of preparation. Fresh-frozen plasma (FFP) may be prepared by apheresis (FFP apheresis, FFPA, or apheresis FFP, AFFP) or whole blood processing and must be frozen within 8 hours of phlebotomy in Canada [41] and the United States [42]; in Australia, freezing of FFPA must commence within 6 hours of collection and that of FFP within 18 hours of collection [43]. In Europe, plasma may be labeled as FFP if it is separated from whole blood and frozen within 6–18 hours of phlebotomy and provided that it contains ≥0.70 IU FVIII activity/mL; whole blood may also be used for FFP manufacture for up to 24 hours after phlebotomy, provided that it is rapidly cooled and held at 20 to 24°C and that it satisfies the FVIII activity requirement [10]. Frozen plasma (FP, also known as FP24 or PF24) must be frozen within 24 hours of phlebotomy and is employed in Canada and the United States. Canada specifies that 75% of units tested must contain ≥0.52 IU FVIII activity/mL and that 1% of monthly site production must be tested [41]; neither American government regulations nor AABB standards specify required activity levels. The United States Food and Drug Administration (FDA) recognizes two forms of FP: PF24 and PF24RT24 (plasma frozen within 24 hours after phlebotomy held at room temperature up to 24 hours after phlebotomy). PF24 must be refrigerated or be made from whole blood refrigerated within 8 hours of phlebotomy and frozen within 24 hours of phlebotomy. PF24RT24 must be made from apheresis devices, whereas PF24 may be manufactured using either whole blood processing or apheresis.

In general, FP, PF24, and PF24RT24 (collectively called FP-type plasma in this review) may show diminished levels of some labile coagulation proteins compared to FFP. Most recommendations concerning plasma transfusion do not stipulate a difference in indications between FFP and FP-type plasma, except where these products are transfused for the treatment of individual deficiencies of labile clotting factors such as FV, FVIII, Protein C, and Protein S. In such instances, FFP is favoured over FP-type plasmas as they have been shown to have lower levels of labile factors. Given the widespread availability of FVIII concentrates and the availability of Protein C concentrates in some jurisdictions, operational differences between FFP and FP-type plasmas reduce to a theoretical preference for FFP in FV and Protein S deficiencies alone.

Those regulatory bodies that focus on FVIII activities presumably do so because FVIII is the most labile of the coagulation factors. However, the connection between FVIII activity levels and the ability of transfused plasma to restore hemostasis is dubious. Firstly, FVIII appears to be present in considerable excess in healthy individuals; reductions below 0.4 IU/mL FVIII activity are needed on a chronic basis to show any clinical signs of pathology [44]. Secondly, FVIII is an acute phase reactant [45] whose levels are expected to rise in injury or disease. Thirdly, FVIII levels in massively transfused surgical patients decline less rapidly than other factors as the number of transfused red cell units increases [34].

Cryoprecipitate is a blood product derived from frozen plasma by slow thawing at refrigerated temperatures. Under these conditions large adhesive proteins reversibly precipitate and are captured in a small volume following expression of cryosupernatant. Historically cryoprecipitate served as a source of FVIII/VWF, but it is now employed primarily as a source of fibrinogen where fibrinogen concentrates are not available or not nationally indicated. Plasma quality indirectly affects the quality of cryoprecipitate, which remains in use in the USA, UK, Canada, and Australia, but which has been replaced by fibrinogen concentrates in much of Western Europe [46]. UK regulations require that 75% of units tested contain >140 mg fibrinogen and 70 IU/mL FVIII [47], Canada mandates at least 150 mg of fibrinogen per unit [48], the FDA stipulates a minimum of 150 mg fibrinogen and 80 IU FVIII per bag [46], and Australia specifies the same content of fibrinogen and FVIII as the UK, as well as a minimum of 100 IU VWF per unit [43].

### 2.7. Measures of Plasma Quality

Plasma quality is currently assessed in vitro, using coagulation factor and coagulation-related protein assays. Investigators have employed single factor assays, more global hemostasis tests, thrombin generation, or viscoelastic approaches to characterize different forms of plasma.

#### 2.7.1. Coagulation Factor Assays

Most investigators addressing plasma quality issues have employed one-stage coagulation tests. In these assays, anticoagulated plasma samples are recalcified in the presence of factor-depleted plasmas, with coagulation being initiated by the addition of either tissue factor and anionic phospholipids (prothrombin time- (PT-) based assay) or a silicate or other negatively charged polymers combined with anionic phospholipids (activated partial thromboplastin time- (APTT-) based assay). The time to form a clot in such assays is then measured, and factor activity levels in the test plasma are correlated to standard plasma sample clotting times. Most investigators in this field employ automated coagulation analyzers.

#### 2.7.2. Hemostasis Screening Tests

Two in vitro clotting tests are widely used by hematologists to screen patients for bleeding disorders or to monitor drug therapy: the PT and the APTT [49]. These tests use the initiators of coagulation defined above for the coagulation factor assays, but simply on recalcified plasma. Both have also been employed in investigations of plasma quality. However, these tests are typically insensitive to reductions in single coagulation factor activity of less than 50%. The APTT is best suited to the initial investigation of suspected hemophilia or the monitoring of heparin or heparinoid drugs used to counter thrombosis. The PT is best suited to monitoring drugs like warfarin that disrupt vitamin K antagonism and reduce the functionality of vitamin K dependent proteins, for antithrombotic benefit. The PT has been standardized as the international normalized ratio (INR), which compares patient PT clotting times to the geometric mean of a group of healthy controls of both genders and which includes a factor related to the potency of the tissue factor preparation used to initiate the test. Both APTT and PT may be performed rapidly as they can be completed in less than one minute per sample, but several commentators have noted that the use of these tests to guide transfusion practice constitutes a use for which neither was designed [50].

#### 2.7.3. Thrombin Generation

The thrombin generation assay (TGA) relies on thrombin cleavage of a fluorogenic substrate to follow changes in thrombin levels in test plasma during a 30- to 60-minute period. It is initiated with either low concentrations of tissue factor for extrinsic pathway activation or negatively charged biopolymers such as ellagic acid or kaolin for intrinsic pathway activation and is accelerated through the inclusion of anionic phospholipids. TGA features a lag phase, a period of increasing thrombin generation, and a period during which the amount of thrombin being generated declines back to baseline. Time to peak, peak thrombin concentration, and endogenous thrombin potential (ETP, the area under the thrombin versus time curve) are typical calculated parameters. For plasma quality determination, the test is performed in plasma rather than whole blood. Although useful for relative within study comparisons, such as before and after manipulations such as pathogen reduction, TGA is currently viewed as being too variable for routine clinical use, a criticism that may have implications for use in plasma quality investigations [51]. It is certainly a much more time-consuming test than “time to clot” assays.

#### 2.7.4. Viscoelastic Testing

Viscoelastic testing refers to thromboelastography (TEG) or rotational thromboelastometry (ROTEM) [51, 52]. Both technologies record viscoelastic changes during blood or platelet-rich or platelet-poor plasma clotting by following the changes in oscillation of a pin or wire immersed in the test fluid; in TEG the cup is mobile and the pin or wire immobile, while in ROTEM the mobilities are reversed. Either assay can be initiated using kaolin and phospholipids or tissue factor. Test runs can require up to 30 minutes for completion. Both technologies have been used as point-of-care tests to guide hemostatic therapy and have been employed in pivotal trials of fibrinogen concentrates [53].

### 2.8. Manipulations of Donated Blood or Plasma with the Potential to Affect Clotting Activity

Plasma samples for reference range determinations are taken under ideal conditions, in which a small volume of blood is taken from a volunteer into vacutainer tubes and immediately centrifuged, and the plasma is aspirated and snap-frozen for subsequent assay. Transfusable plasma, in contrast, is collected on a larger scale and subjected to additional manipulations, all of which have the potential to decrease one or more coagulation factor activities. Many investigations of plasma quality have focused on the effect of such manipulations, to address the question of whether or not a perceived benefit, such as leukoreduction to reduce transfusion reactions, extended holds of whole blood prior to processing to improve blood operator efficiency, or extended storage of thawed plasma to reduce wastage, is worth the cost of reduced coagulation-related protein activity.

#### 2.8.1. Leukoreduction

Prestorage leukoreduction of whole blood has been implemented for all blood donations in various European countries, in Canada, and by the majority of American blood operators. Benefits include reductions in the frequency and severity of febrile nonhemolytic transfusion reactions (FNHTRs); in the risk of cytomegalovirus transmission; and in the risk of alloimmunization and platelet refractoriness [3]. Cardigan et al. examined five different whole blood filters, using a pool-and-split design, and processed filtered or nonfiltered whole blood into plasma. Some filters had no effect on any tested coagulation factor activity, including fibrinogen or prothrombin; others caused a modest increase in median PT or APTT of less than one second. Filters associated with a reduction in coagulation factor activity decreased median FV, FXI, and FXII activity by 13–20%, with no effect on VWF and only a 5% reduction in median FVIII activity [54]. Heiden et al. reported similar findings, along with the observation that elastase released from neutrophils during leukoreduction was fully neutralized by alpha-1-antitrypsin and that complement-related C3a was found in plasma from filtered blood as inactive C3a-desArg [55]. Most recently Chan and Sparrow split whole blood into pediatric-sized packs and applied 6- or 24-hour holds with or without leukoreduction prior to plasma production [56]. These authors found that the leukoreduction filter they employed partially trapped microparticles and its use was associated with 15–20% reductions in fibrinogen, FVIII, and FXII activities. Leukoreduction slowed clot formation and reduced clot strength as judged by kaolin-initiated thromboelastography; whole blood hold time did not affect these parameters despite the reductions in clotting factor activities.

#### 2.8.2. Holding Effects

Holding blood at either refrigerated or ambient temperatures provides considerable logistical advantages to blood operators, with respect to the ability to collect at remote clinics and to rationalize shift work by processing staff. Effects of hold times on component quality have been reviewed by van der Meer and de Korte [57]. Pietersz et al. reported mean losses of 20% of FVIII activity in whole blood units (*n* = 10) rapidly cooled to 20–24°C and held for 24 hours prior to plasma production via the buffy coat method without leukoreduction, less than that reported in refrigerated whole blood held for 26 hours in a smaller study (*n* = 5) [58]. O'Neill et al. split whole blood units (*n* = 10) into half-units and stored them at 4°C or 22°C for 8 hours and then refrigerated all half-units for the next 16 hours, making plasma from all units at 8 or 24 hours via the platelet-rich plasma (PRP) method. FV, FVII, FX, fibrinogen, and Proteins C and S activities in plasma were unchanged relative to baseline, at collection values [59]. Eight-hour storage reduced mean FVIII activity 13% relative to baseline values, and twenty-four-hour storage reduced it by an additional 20% [59]. Wilsher et al. allocated 80 whole blood donations into four equal groups to assess FFP production within 8 hours or after 24-hour holds at refrigerated or ambient temperatures, finding no effect on fibrinogen activities of any condition, and only a modest loss of FV activity associated with 24-hour ambient temperature hold. FVIII activity losses were limited to 21% relative to <8-hour processing with or without active cooling and were significantly less than the 36% mean reduction in activity seen in 24-hour refrigerated hold units [60]. van der Meer and de Korte also reported no effect of active cooling of whole blood held overnight on FVIII activity in generated plasma [61]. These results on holding effects have in general been replicated by investigators using different whole blood processing methods and different anticoagulants in different nations and transfusion services [36, 62–66]. There is no evidence that the declines in coagulation factor activity associated with holding effects compromise clinical efficacy, and therefore economic and strategic considerations have prompted greater use of FP-type plasmas over FFP [42, 67].

#### 2.8.3. Speed of Freezing

Initial studies on the effect of the speed of freezing of plasma focused on FVIII activity, since efficient recovery of FVIII was of paramount importance in the fractionation industry prior to the advent of recombinant FVIII. Carlebjork et al. noted a positive correlation between the rate of freezing of plasma and the recovery of FVIII activity [68]. Swärd-Nilsson et al. examined 100 mL aliquots of plasma in plastic bottles. FVIII activity in plasma was found to be stable to prefreezing room temperature holds of up to 4 hours. Rapid freezing to a core temperature of −30°C within 60 minutes, as compared to slow freezing over 24 hours, resulted in mean FVIII recoveries of 93.6 versus 68.2% [69]. Runkel et al. pooled and split whole blood donations, generating plasma within 8 hours of phlebotomy that was frozen at the 8-hour time point or held at either ambient or refrigerated temperatures for an additional 16 hours prior to freezing [70]. Freezing was conducted either in slow (transfer to −20°C) or in fast (to −30°C core temperature) modes to imitate FDA or European requirements, respectively. Prolonging the “time to freezer” reduced median FVIII levels ~10%, while combining prolonged time to freezer with slow freezing together reduced median FVIII levels 18.5%. Room temperature storage reduced median Protein S activity by 27%, and prolonged storage at room temperature correlated with a 7% decline in FVII activity. No changes were noted in FV, FXI, fibrinogen, VWF, or Protein C activities. APTT and PT values were modestly increased by prolonged time to freezer and slow freezing. In general, available data suggests that rapid freezing and limiting the time from phlebotomy to freezer best preserve coagulation activity in transfusable plasma, but it does not seem that such steps give any clinical advantage to FFP over FP-type plasmas. Where permitted by regulations, the selection of lower cost slow freezing modalities for transfusable plasma has therefore not been impeded.

#### 2.8.4. Stability of Thawed Plasma for Transfusion

Plasma takes time to thaw and time to infuse. Dose recommendations in the 10–15 mL/kg range translate into 700–1050 mL of plasma for a 70 kg individual or 3-4 units [33]. Two factors spurred interest in the question of how long thawed plasma may be refrigerated prior to use in an effective and safe transfusion: efforts to reduce transfusion-related acute long injury (TRALI) and efforts to facilitate early administration of plasma to trauma patients. Shifts to predominantly male transfusable plasma were made by several blood operators to reduce one factor contributing to TRALI, anti-HLA antibodies in previously pregnant donors [71]. This move put supply pressure on FFP and increased interest in prolonged use of thawed FP-type plasmas. Similarly, concern over the development of trauma-induced coagulopathy and a perceived need to start plasma transfusion as soon as possible led many trauma centres in the industrialized world to adopt massive transfusion protocols, involving, in part, the maintenance of thawed inventory of AB-universal donor plasma [72].

Downes et al. carried out a small study (*n* = 5 for each of 3 ABO blood groups) of thawed FFP stability after AABB standards expanded its shelf-life from 24 to 120 hours. No significant differences from day 1 to day 5 activity values were noted for FII, FV, FVII, FX, or fibrinogen. FVIII values dropped 35–41% during the storage period, with the majority of the decline occurring in the first 24 hours of storage [73]. Scott et al. examined 20 FFP units at thaw or after 120 hours of refrigerated storage, noting a 47% drop in FVIII activity as well as losses of FV and FVII activity of 21 and 33% and smaller losses of 3–8% in FII, FX, VWF, and Protein S activities [74]. Scott et al. also examined the stability of 14 FP24 units, noting similar patterns; the largest losses in this product were for FV, FVIII, VWF, and Protein S (31, 28, 17, and 15%, resp.) [74]. Sheffield et al. probed the stability of Canadian thawed FP (*n* = 54), produced using the buffy coat method, finding it noninferior in residual FVIII activity at 120 hours to the 0.48 ± 0.12 IU FVIII/mL reported by Sheffield et al. from PRP method FP24; mean losses of 20, 14, and 41%, in FV, FVII, and FVIII, respectively were observed, with no alteration in fibrinogen activity and a 9% prolongation of PT by 120 hours [37]. Similar results were obtained in stability studies of FFPA: Sidhu et al. found FV and FVIII decreases of 9 and 14% after 120 hours of refrigerated storage and no change in FII, FVII, FX, FXI, and fibrinogen (*n* = 20, sodium citrate anticoagulant) [75]; and Von Heymann et al. noted losses of FII, FV, FVII, FVIII, FIX, FX, and FXI ranging from −8% (FII) to −47% (FVIII) (*n* = 20, acid citrate dextrose [ACD] anticoagulant) after 144 hours of refrigerated storage [76]. Cookson et al. observed mean losses of 11% FV and 33% FVIII activities relative to baseline values and lesser declines of FII, FVII, FIX, and FXII after 144 hours of refrigerated storage of thawed FP made from whole blood held at room temperature for 24 hours. These factor losses failed to affect thrombin generation and produced only marginal lengthening of clot time, but no change in clot strength, in viscoelastic testing [77].

These data have been interpreted in different ways in different jurisdictions, likely due to the lack of clinical data with which to anchor them to outcome measurements. American and Canadian regulators permit the transfusion of refrigerated thawed plasma stored for up to 120 hours, whereas the Council of Europe requires transfusionists to administer the product as soon as possible after thaw, and in the United Kingdom 24-hour refrigerated storage is permitted [78].

### 2.9. Pathogen-Reduced Plasma

Plasma may also be subjected to additional manipulations designed to increase the considerable protection already afforded to patients by its prerelease immunological and nucleic acid testing for transfusion-transmitted pathogens [5]. These include treatments of pooled plasma with solvent-detergent mixtures [79] and of individual plasma units with agents such as methylene blue or amotosalen, which must be removed prior to infusion after illumination of units with ultraviolet or visible light, or riboflavin (vitamin B2), for which there is no removal requirement [80]. Quality assessment of these products has been largely limited to comparisons to the FFP from which they are derived. Efficacy determinations have included clinical studies, including those leading to licensure on the basis of similar performance to FFP.

#### 2.9.1. Pathogen Reduction of Pooled Plasma

Solvent-detergent plasma (SDP) is a pooled plasma product subjected to solvent (tri(n-butyl)phosphate) and detergent (Triton X-100) treatment to destroy enveloped viruses; prior to filtration, bagging, and freezing, the solvent and detergents are removed by oil and solid phase extraction steps [79]. The process was originally invented by scientists at the New York Blood Center [81]. Although several SDP are licensed in different countries, most of the information concerning SDP in the public domain relates to Octaplas, the SDP product manufactured by Octapharma GmbH (Vienna, Austria), which has been in continuous clinical use in Europe since 1992, and elsewhere for lesser periods of time. Unpaired comparisons of 12 consecutive batches of Octaplas and 12 random quarantine FFP units revealed no significant differences in FII, FV, FVIII, FIX, FX, FXII, FXIII, VWF, antithrombin, or Protein C activities [82]. Minor reductions in fibrinogen and FVII were noted, and substantial reductions in mean Protein S activity (38%) and alpha-2 antiplasmin (78%) were also observed. In 2009 the manufacturing process for Octaplas was modified to include a prion reduction affinity chromatography (“liquid gel”) step (Octaplas LG). After implementation of this change, no substantial differences were noted between batches of the first or second generation product by coagulation factor assays, thrombin generation, or viscoelastic testing [83].

#### 2.9.2. Pathogen Reduction of Single Plasma Units

Three technologies, licensed in at least some parts of the world, are available for pathogen reduction (PR) of single plasma units [80]. All rely on photoactivation by visible or ultraviolet light and the addition of chemicals to plasma or PRP: methylene blue (MB); amotosalen (AS); or riboflavin (RF). All attack pathogen nucleic acids in different ways, reducing infectivity by many orders of magnitude. All lead to some reductions in coagulation factor content as a potential cost of increased safety. These effects have been most clearly demonstrated in paired comparisons of FFP, MB-FFP, and AS-FFP. Osselaer et al. found that losses of fibrinogen, FV, FVIII, FXI, and Protein S activities were greater for MB-FFP than AS-FFP, although the most affected proteins, fibrinogen and FVIII, still retained 70–80% of their FFP activity levels after treatment and freezing and thawing of MB-FFP. The authors noted that the mean values for all 18 plasma protein activities tested fell within normal references ranges in all cases and that ADAMTS-13 was unaffected by either PR treatment [84]. Backholer et al. reported similar findings in another paired comparison in which FV, FXI, FXIII, and fibrinogen losses were significant for MB-FFP, but not for AS-FFP, by analysis of variation (ANOVA) of FFP, MB-FFP, and AS-FFP; six other coagulation factors were unaffected by either treatment [85]. Cardigan et al. noted reductions of 10% in ETP and 30% in peak thrombin in thrombin generation assays of paired MB-FFP versus FFP, but no effect on clot formation rate and a surprising 20% increase in clot firmness by ROTEM [86]. Hubbard et al. noted alterations in fibrin clot structure for both AS-FFP and RF-FFP [87].

Hornsey et al. examined the recovery of coagulation-related proteins before and after production of RF-FFP (*n* = 20) [88]. While Protein S and antithrombin were unaffected by riboflavin treatment, ten other proteins exhibited diminished recoveries, with the lowest recoveries being noted for fibrinogen, FVIII, FXI, and ADAMTS13 (68–79%). FVIII activity levels nonetheless met Council of Europe requirements, at 0.76 IU/mL in the study, as did all other factors.

### 2.10. Dehydrated Plasma

Plasma was originally introduced into clinical practice, during World War II, as a lyophilized product appropriate for battlefield use [40]. Such dehydrated plasma formulations attempt to replace conventional plasma with a product that does not require a cold-storage chain and which can be reconstituted more rapidly than frozen plasma can be thawed. Plasma may be dried either in pools (with or without pathogen inactivation) or as single units. The most common approach is freeze-drying, or lyophilization, a process by which plasma is rapidly frozen and maintained at low temperatures under partial vacuum. Sufficient heat is then introduced such that frozen water in the product is driven off via sublimation. An alternative approach involves spray drying, in which atomization and heat are used to evaporate microdispersed water droplets. Both technologies provide stable, reversibly dried products in which >95% of the original water content is removed [89].

The two best characterized freeze-dried plasma (FDP) products licensed for use in Western countries are French lyophilized plasma (PLyo) [90] and LyoPlas N-w, produced by the German Red Cross Blood Service West [91]. The former product is made from a pool of A, B, and AB pathogen-inactivated FFPA plasmas from no more than 10 donors, with each unit containing ≥0.9 IU/mL FVIII activity; the latter is made from a single unit of quarantined plasma. Quarantined plasma is not released for manufacturing until the donor has returned to the blood or plasma centre in good health and tested negative for infectious disease markers a second time.

Martinaud et al. tested aliquots of 24 batches of PLyo before and after lyophilization [92]. Fibrinogen, FXI, FXIII, Proteins C and S, antithrombin, and alpha-2 antiplasmin were unaffected by the process. Factors V and VIII exhibited lyophilization-dependent losses of 20–25%. No differences were found between the two kinds of plasma in thrombin generation assays initiated with 5 pM tissue factor and no differences were found in viscoelastic testing.

Bux et al. described aspects of the quality of LyoPlas N-w, which is the single donor, quarantined plasma-derived German FDP [91]. Compared to the thawed FFP from which the product was made, lyophilization had no effect on fibrinogen, Protein S, or antithrombin activities. Ten to 25% losses in activity were noted for factors V, VIII, XI, vWF, and plasminogen, as well as a 10% prolongation in aPTT. This product has been extensively used in both military and civilian settings; from 2007 to 2011 more than 230,000 units were delivered to hospitals and doctors' offices and provided to the Germany Army. However, no true measures of clinical efficacy appear to have been reported in the public literature.

### 2.11. Fractionation versus Transfusion of Plasma

Plasma does not have to be transfused to be medically useful. Recovered or source plasma can be fractionated into purified plasma protein products such as immunoglobulin or coagulation factor concentrates [93]. Immunoglobulin therapy clearly benefits some patients, such as those with primary or secondary immune deficiency, immune thrombocytopenia, and chronic inflammatory demyelinating polyradiculoneuropathy [94]. vWF [95] or fibrinogen concentrates benefit patients with deficiencies of these factors [96], and prothrombin complex concentrates reverse warfarin therapy more rapidly than plasma transfusion with respect to laboratory values and more effectively with respect to restoration of hemostasis [97]. Fibrinogen concentrates and PCCs may provide superior therapy compared to plasma in bleeding patients without genetic deficits due to the possibility of rapid restoration of hemostasis and are under active investigation. Even in TTP, a recombinant ADAMTS13 is in preclinical development, a product that may in future supplement the most evidence-supported indication for plasma transfusion [98]. Given the paucity of evidence of benefit for the medical use of transfusable plasma and the existence of high-level clinical evidence of efficacy for some of the indications for which fractionated products are employed, it is likely that an increasing proportion of plasma will be fractionated rather than transfused.

### 2.12. Appropriateness of Plasma Transfusion and Utilization Trends

The amount of plasma transfused in several countries has declined in recent years, including Canada, the United Kingdom, and the United States [99, 100]. The decline has been linked to efforts to increase appropriateness of plasma transfusion, to the increased availability of plasma protein products such as PCCs, and to improvements in surgical techniques. It seems likely that this trend will continue, barring new clinical data supporting plasma transfusion.

Although there is disagreement among physicians as to the value of plasma transfusion and some indications are supported by weaker evidence than others, there is reasonable expert consensus as to uses of transfusable plasma which are inappropriate [33]. Multiple audits of transfusable plasma utilization have shown that over 30% of the time, plasma is transfused inappropriately, typically in efforts to alter a mildly elevated INR and to nonbleeding patients [101–104]. Although most studies of plasma quality end with an exhortation to trialists to obtain better RCT data, it is likely that improvements in plasma utilization could be achieved more readily by diminishing inappropriate transfusion of plasma.

### 2.13. Future Prospects for Quality Assessment of Transfusable Plasma

An extensive literature exists regarding the effect of different manipulations or process changes on coagulation factor activities in transfusable plasma or its derivatives. However, linking these changes to differences in overall hemostatic function of plasma has not been extensively attempted. Available data using more global tests of hemostasis such as thrombin generation and viscoelastic testing has started to suggest that many of the observed alterations in one or more coagulation factors are not particularly relevant to hemostasis, given its complexity and the number of mechanisms that can combine to adapt to changes in procoagulant and/or anticoagulant protein profiles within the large functional reserve of this biological fluid. If thrombin generation and viscoelastic tests can be better correlated with patient clinical status and adapted to more rapid execution, such assays may supplant coagulation factor assays in the effort to answer the elusive question, “is this unit of transfusable plasma of high quality?”

## 3. Quality of Platelet Concentrates

### 3.1. New Modes of Platelet Storage: What Should We Be Measuring?

Platelet transfusions are essential for the treatment of patients with acute bleeding or hemorrhage and for prevention of bleeding in severe thrombocytopenia [105–107]. When the vasculature is damaged, platelets adhere, aggregate, and become activated, eventually forming a platelet plug, as well as providing a catalytic surface for thrombin generation. Platelet concentrates (PCs) for transfusion are typically prepared either from whole blood or by apheresis and are stored at RT (20–24°C) for up to 7 days. The shelf-life of platelets stored at RT is limited due to the risk of bacterial growth and contamination, which can cause life-threatening transfusion-related infections [108]. Additionally, platelets stored at RT gradually deteriorate and undergo a decline in hemostatic and metabolic function, which is known collectively as the platelet storage lesion [109–112].

Due to the short shelf-life of PCs, providing platelets to remote, rural, and austere environments is often challenging. Alternative storage modalities such as cryopreservation and cold or refrigerated storage are currently being explored to overcome the challenges associated with the short shelf-life of PCs. It is clear that platelets stored under these conditions appear to be very different from standard, RT-stored platelets, when assessed using in vitro assays previously applied to RT-stored platelets alone [113–115]. Further, cryopreservation of platelets is a relatively new field, and while cold storage of platelets has been studied for many decades, there is a renewed interest in this storage mode, and the in vivo efficacy of these novel platelet components is only now being investigated. Standard measures of activation and metabolism are not always sufficient for cold or cryopreserved platelets and generally do not correlate well with in vivo transfusion outcomes. A well-defined panel of assays to measure the in vitro quality of these components has not yet been established. This section of the review will explore the differences between platelets stored frozen, refrigerated, or at RT and will address the techniques that may be most appropriate to measure their in vitro function and quality.

### 3.2. Assays for In Vitro Assessment of Room Temperature-Stored Platelets

Many techniques can be used to measure platelet function, metabolism, and quality in vitro. The simplest measurement is platelet swirl, whereby platelets with a discoid shape refract light and hence appear to swirl when exposed to a light source, whereas platelets that have lost their discoid morphology do not swirl [116]. Hypotonic shock response (HSR) is based on the ability of platelets to extrude water when placed in a hypotonic solution, is reflective of an intact membrane, and typically declines during ex vivo platelet storage [117–119]. Extent of shape change (ESC) is a photometric measurement of platelet shape change in response to agonists such as ADP and an indicator of the ability of platelets to maintain their discoid morphology. As is the case with HSR, the ESC response of platelets declines during storage [117, 120, 121]. Lactate dehydrogenase (LDH) release into the supernatant can be measured as an indicator of platelet lysis and loss of viability [122, 123], often accompanied by a reduction in platelet count and swelling, measured by an increase in mean platelet volume (MPV).

Platelets produce energy through two major metabolic pathways: anaerobic glycolysis in the cytoplasm and oxidative phosphorylation in the mitochondria, which contributes approximately 80% of platelet ATP [124]. Therefore, measurement of glucose consumption, lactate production, and changes in pH during platelet storage are widely used as indicators of active metabolism, as are measurements of pO_2_, pCO_2_, and HCO_3_ [123, 125–128]. Platelet surface markers are also indicative of platelet activation, including CD62P (P-selectin) and exposure of phosphatidylserine (PS) measured by annexin V binding, and are typically measured by flow cytometry [129, 130]. As platelets become activated, the contents of their granules, such as RANTES, CD40L, soluble CD62P, and platelet factor 4 (PF4), are released and accumulate in PCs during storage [122, 123, 129–131].

As part of the platelet storage lesion, platelets also lose their ability to respond to agonists such as ADP, collagen, and thrombin, which can be measured photometrically using light transmission aggregometry [126, 132–134]. However, the loss of ability to respond to agonists is reversible, and platelets have been shown to recover their capacity to respond to agonists following transfusion [135]. Similar instrumentation can be used to measure HSR and extent of shape change ESC. Assays that measure platelet hemostatic function and procoagulant capacity can also be applied to RT-stored platelets. These will be discussed in more detail in subsequent sections.

Despite the wide variety of parameters that can be measured in vitro, they do not always correlate with in vivo measurements. The most commonly used in vivo measurements are platelet recovery and survival following transfusion, as mandated by the FDA (66.7% recovery of the same subjects fresh platelets after 24 hours and 58% recovery of the same subject's fresh platelets) [136]. However, platelet components in routine clinical use can fail to meet these criteria, highlighting a need for alternative criteria that provide information regarding platelet function following transfusion [137–140]. Nonetheless, there are strong correlations between some in vitro measurements and platelet recovery and survival in vivo. For example, lactate concentration has been shown to correlate strongly with reduced in vivo recovery and survival following transfusion. A similar correlation was observed for pH, which itself is influenced by lactate production [141]. Increases in markers of activation, such as expression of CD62P (P-selectin) and annexin V binding, have also been strongly correlated with reduced in vivo recovery [141, 142]. More recently, metabolomics has been used to better understand interdonor variations and identify metabolites that may be markers of high or low in vivo platelet recovery and survival [143]. However, the outputs of such studies are complex, and interpretation relies on powerful bioinformatics algorithms, rendering metabolomic testing not yet applicable for routine testing laboratories assessing platelet quality.

### 3.3. Alternative Modes of Platelet Storage

To circumvent problems associated with the short shelf-life of platelets, alternative modes of platelet storage have been investigated. These include platelet cryopreservation, cold storage, and lyophilization. Here we focus on cryopreservation and cold platelet storage, as these are being most actively pursued by several groups. Platelet cryopreservation typically involves addition of DMSO to a final concentration of 4–6% (vol/vol) [138, 144, 145] and storage at −80°C for between 2 and 4 years [146–148]. Platelet cryopreservation was pioneered by the US military and later the Netherlands military, who have successfully transfused over 1000 cryopreserved platelets during military deployments to Bosnia, Iraq, and Afghanistan [149, 150]. This technology is now being adopted by many countries. Platelet cryopreservation and the subsequent thawing processes are, however, time consuming and more expensive than standard RT storage, and other alternatives have been sought. One such alternative is cold storage of platelets, which is extremely simple logistically, as such platelet products can be stored in a refrigerator as per red cells and do not require agitation.

### 3.4. Platelet Cryopreservation

Methods used today for platelet cryopreservation were pioneered by Handin and Valeri for the US military and first published in the 1970s [151]. The original protocol described addition of 5 to 6% DMSO to hyperconcentrated platelets prepared from a single unit of whole blood using the PRP method [151, 152]. The DMSO was not removed prior to freezing, and after thawing the platelets were washed and resuspended in autologous plasma. The platelets were found to be hemostatically effective upon transfusion [153]. Subsequent protocols described removal of DMSO prior to freezing, as this allowed platelets to be thawed and reconstituted immediately after thawing with reduction of DMSO content [144]. This process was adopted by the Netherlands Military Blood Bank [149] and many countries including the USA, Brazil, France, Turkey, and Singapore now have programs to implement cryopreserved platelets for civilian and/or military use, with similar cryopreservation protocols [138, 154, 155].

In vitro data from early studies showed changes in platelet shape and structure following cryopreservation [156]. Further studies have subsequently found that cryopreserved platelets have impaired HSR and aggregation responses to ADP, epinephrine, and collagen, as well as reduced oxygen consumption; moreover platelet activation of a high proportion of cryopreserved platelets was suggested by increased P-selectin and elevated annexin V binding to PS on a high proportion of platelets, indicative of poor in vitro quality and function [115, 138, 144, 157, 158].

Despite these impairments, cryopreserved platelets were shown to correct bleeding time in patients with hematological disorders when transfused together with other blood products [159] and to increase posttransfusion platelet counts and correct bleeding times when cryopreserved HLA-matched allogeneic or autologous platelets were used for treatment of thrombocytopenic alloimmunized patients [160, 161]. More recent investigations have demonstrated similar efficacy [162].

A randomized controlled comparison of cryopreserved and liquid-stored platelets demonstrated that cryopreserved platelets were potentially more effective than fresh, liquid-stored platelets for controlling surgical bleeding. Although the platelet count increment was lower in patients receiving cryopreserved platelets, blood loss and the number of postoperative blood products transfused were lower in this group, suggesting that despite increased clearance, cryopreserved platelets may in fact be superior to conventional liquid-stored platelets for controlling bleeding [163]. Despite in vitro deficiencies and increased activation, the platelets were still effective upon transfusion, supporting a hypothesis that despite being already activated, other changes in cryopreserved platelets rendered them more procoagulant and potentially more hemostatically active. The findings from this study also highlight the lack of correlation between in vitro and in vivo function in cryopreserved platelets, and hence the need for characterization with an extended suite of platelet assays.

More recent studies using techniques to measure the hemostatic function of platelets in vitro have expanded upon these earlier studies, suggesting they may be more appropriate measurements for cryopreserved platelets. These include techniques such as thrombin generation (see Section 2.7.3 above) and viscoelastic testing (see Section 2.7.4 above), which permit measurement of the procoagulant activity of both platelets and platelet microparticles. Consideration must also be given to enumeration of microparticles, as they contribute to the procoagulant function of cryopreserved platelets and are abundant in high numbers after thawing [115, 164, 165]. It should also be noted that no one test addresses all aspects of platelet quality and function, and several should be used in combination.

Thromboelastography (TEG) was developed to measure the clot formation and strength in whole blood and is now used routinely for evaluation of coagulation dysfunction in patients. TEG provides information regarding time to clot formation, clot strength, and fibrinolysis through continuous measurement of viscoelasticity during all stage of clot development and dissolution [166]. Rotational thromboelastography (ROTEM) provides similar information. TEG and ROTEM can be used to assess platelet function and storage-associated changes in liquid-stored PCs, as R-time (clot reaction time) and K-time (time for clot amplitude to increase from 2 to 20 mm) decrease during storage [167, 168]. TEG and ROTEM measurements are also suited to evaluation of cryopreserved platelets, which have a reduced R-time compared to liquid-stored platelet, with reduced clot strength [115]. Similar findings have been observed with ROTEM and are supported by perfusion studies under shear stress, whereby cryopreserved platelets adhere to surfaces and become activated to form stable aggregates with fibrin deposition [169].

Thrombin is a critical component of the coagulation cascade, and generation of thrombin is reflective of hemostatic function. Thrombin generation in platelet concentrates can be assessed using calibrated automated thrombinography (CAT) [170]. PS and tissue factor, both of which are present in high amounts on the membrane of cryopreserved platelets and their microparticles, have the potential to trigger thrombin generation [115, 171, 172]. Thus thrombin generation is useful for measuring the procoagulant activity of cryopreserved platelets and reveals that cryopreserved platelets and platelet microparticles are also more procoagulant than fresh, liquid-stored platelets in terms of the amount of thrombin they generate and the lag time for thrombin generation [115, 171]. However, the influence of the solution used for reconstitution after thawing must be taken into consideration, as non-plasma-based reconstitution solutions may not provide sufficient tissue factor for detectable thrombin generation [115].

CD62P (also known as P-selectin) is a well-characterized marker of platelet activation, which increases when platelets are stored at room temperature [122, 123, 173]. Given that cryopreserved platelets are known to be activated, it would be expected that CD62P expression would be high on cryopreserved platelets. However, there are variations in what has been observed by different groups examining cryopreserved platelets, whereby some have observed high expression (>60%) [138, 171], some have observed partial activation with 20–40% CD62P expression [162, 165], and others have observed low CD62P expression [145, 174, 175]. This variation may arise due to differences in the platelets themselves, differences arising from use of different antibody clones, staining protocols, or instrument settings on different flow cytometers. Exposure of PS on the outer leaflet of the platelet membrane indicates platelet activation and increases the procoagulant response of platelets [176]. Cryopreserved platelets also express high levels of PS and shed more PS-positive platelet microparticles when compared to fresh liquid-stored platelets. Annexin V binding to PS may therefore be a better indicator of platelet activation in cryopreserved platelets, as it is consistently reported as high by many groups [115, 145, 158, 171].

PS on microparticles acts as a catalytic site for the FXa/F Va complex, accelerating thrombin formation, giving them procoagulant activity [115, 177]. The contribution of PS to procoagulant activity can be measured in clot-based or chromogenic assays that provide functional measurements of procoagulant phospholipids [178].

Platelet microparticles are best measured by flow cytometry, so that expression of a platelet-specific antigen such as CD61 may be combined with annexin V for confirmation of PS externalization, as part of the membrane remodelling that occurs during microparticle generation [179, 180]. The choice of surface markers for quantitation and characterization of platelet microparticles generated during cryopreservation is also of importance, as the proportionate expression of platelet surface markers is very different from marker expression on microparticles found in liquid-stored platelet concentrates [177]. The resolution of the flow cytometer is often a limitation in accurately identifying microparticle populations, as resolution is usually limited to 400–500 nm. However, development of newer flow cytometry instrumentation allows quantification and characterization of microparticle populations with high resolution ranging from 1 *μ*m to 20 nm [181]. Dynamic light scattering can also be used to measure platelet particle size and the relative proportions of particles, although it does not provide information regarding the phenotypic characteristics of the microparticle populations [177].

There is still a paucity of published clinical data on patient outcomes following transfusion of cryopreserved platelets, rendering it difficult to correlate in vitro activity with efficacy or safety. Furthermore, recovery of autologous platelets at 24-hour posttransfusion may not be an ideal measure for cryopreserved platelets, as it does not take into consideration their procoagulant phenotype and their potential to stem active bleeding or hemorrhage more effectively. Rather, inclusion of outcomes such as posttransfusion blood loss, bleeding time, volume, and number of transfusions in clinical studies may be more informative, and subsequent correlation of in vitro function with these outcomes may advance this field. Animal studies using wound or hemorrhage models may also help to bridge this knowledge gap.

### 3.5. Cold Storage of Platelets

Platelets were stored in the cold (at ~4°C) until 1969, when transfused platelets were shown to be more rapidly cleared from the circulation than platelets stored at RT [182]. The majority of platelet transfusions are given prophylactically to thrombocytopenic hematooncology patients [183]. As such, a longer lifespan in the circulation is desirable, and cold storage of platelets ceased, with adoption of storage at RT (20–24°C). However, the advantages of cold storage include prolonged shelf-life due to reduced metabolism, enhanced hemostatic activity, improved bacteriologic safety, and ease of storage and transport, as the same infrastructure for storage and transport of red cells could be utilized.

Platelets stored at 4°C undergo many ultrastructural and metabolic changes, known as the cold-storage lesion. Most notably, cold-stored platelets undergo a shape change from disc to sphere, with an increase in MPV and loss of swirl, mediated by changes in localization of cytoskeletal proteins [113, 184]. Other changes include clustering of the GPIb*α* subunit of the von Willebrand receptor complex on the platelet surface [185, 186] and changes in glycosylation patterns with loss of sialylation and exposure of galactose residues that ultimately leads to clearance by hepatic macrophages through Ashwell Morell receptors [187, 188].

A renewed interest in cold storage of platelets has shown that, as with cryopreserved platelets, cold storage leads to increased activation and more procoagulant function, with increased expression of activation markers such as CD62P and exposure of PS, improved in vitro TEG and ROTEM responses, more vigorous aggregation responses, and increased thrombin generation in comparison to room temperature-stored platelets [113, 114, 189–191]. As such, assays for measurement of in vitro function that are suitable for characterization of cryopreserved platelets, including TEG or ROTEM, CAT, microparticle enumeration, and flow cytometry, are also very useful for characterization of cold-stored platelets.

Some measurements that are less informative for cryopreserved platelets, such as glucose metabolism, are, however, more appropriate for examining differences between cold-stored and RT-stored platelets. For example, cold storage of platelets slows glycolysis as well as attenuating acetate metabolism, possibly through inhibition of oxidative phosphorylation. Given that cold storage causes a change from discoid to spherical shape, measurements such as MPV, swirl, HSR, and ESC are also informative [113, 114, 192].

A shelf-life for cold-stored platelets has not yet been rigorously defined, although FDA approval has recently been given for the narrowly defined instance of administration of cold-stored apheresis platelets to actively bleeding patients, provided the product has been refrigerated for less than 72 hours after phlebotomy [193]. Further investigations with cold-stored platelets under different storage conditions, such as in additive solutions versus plasma, are necessary to generate data that will help establish optimal storage conditions and determine whether a shelf-life beyond that of room temperature-stored platelets is feasible.

Cryopreservation and cold storage lead to many changes in platelet metabolism, surface receptor expression profiles, membrane and cytoskeletal structure, and importantly hemostatic function, some of which may be considered adverse and some advantageous. Measurement of these changes as they occur in vitro and the ability to correlate them with appropriate transfusion outcomes are essential for defining the appropriate indications for these novel platelet components and embedding them into routine use.

## 4. Overview: Quality Assessment of Stored Red Cell Concentrates

Transfusion of red blood cell concentrates (RCCs) is a necessary, lifesaving clinical therapy. RCCs are given to increase oxygen delivery to tissues in clinical situations where the circulating red blood cell (RBC) level is low (anemia) due to RBC loss (trauma/surgical hemorrhage), decreased bone marrow production (chemotherapy, aplastic anemias), defective hemoglobin (hemaglobinopathies, thalassemias), or decreased RBC survival (hemolytic anemias). Approximately 1.2 million RCCs are collected and transfused each year in Canada [194, 195], and more than 90 million units are transfused globally [196]. RCCs are used across a variety of medical and surgical situations with approximately 30% of critical care patients and more than 50% of cardiac surgery patients receiving blood products during their hospital stay [197, 198].

To ensure that RCCs are produced in a consistent and controlled manner, blood collection agencies routinely test products as part of their quality assurance programmes and as part of their continuous improvement activities prior to making changes to equipment or processes. Acceptable standards for product safety and quality are outlined in government regulations and by standard-setting organizations. Despite these stringent control mechanisms, a great deal of variability still exists within the blood components transfused which may affect patient outcomes. Motivated by a need for more concerted efforts to understand the factors affecting RCC quality, this section will review the changes that occur to RBC during blood banking, current quality testing practices, and the factors which affect the quality of stored RCCs.

### 4.1. RBC Physiology and the Storage Injury

The primary function of RBCs is to transport oxygen from the lungs to the body tissues, where the exchange for carbon dioxide is facilitated through the synergistic effects of hemoglobin, carbonic anhydrase, and band 3 protein, followed by carbon dioxide delivery to the lungs for release. Successful oxygen transport is dependent on the efficacy of three critical elements: the RBC membrane, hemoglobin, and the cellular energetics.

The RBC membrane is a fluid structure composed of a semipermeable lipid bilayer with an asymmetrically organized mosaic of proteins. Membrane lipids compromise approximately 40% of the RBC membrane mass, with equimolar quantities of unesterified cholesterol and phospholipids, and small amounts of free fatty acids and glycolipids [199]. Membrane proteins comprise approximately 52% of the RBC membrane mass and can be categorized into integral and peripheral proteins according to their location relative to the lipid bilayer [199]. Integral membrane proteins, such as glycophorin and band 3 protein, transverse the membrane and function as receptors and transporters. In contrast, peripheral proteins are only found on the cytoplasmic surface of the membrane and form the RBC cytoskeleton. The major components of the RBC cytoskeleton are spectrin, ankyrin, protein 4.1, actin, and adducin, which form a mesh-like network of microfilaments that strengthens the RBC membrane while maintaining RBC shape and stability [200]. The unique characteristics of the RBC membrane and cytoskeleton afford the cells the ability to reversibly deform as they traverse the microvasculature and thereby deliver oxygen from the lungs to the tissues.

The second element that has to be maintained for the cells to function normally is hemoglobin. Hemoglobin is a conjugated protein consisting of two pairs of globin chains and four heme groups, each containing a protoporphyrin group and an iron molecule in the ferrous form [201]. The uptake and release of oxygen by the hemoglobin molecule are controlled by the RBC organic phosphate 2,3-disphosphoglycerate (2,3-DPG), which binds to the cleft between globin chains, resulting in a deoxyhemoglobin conformation that facilitates the release of oxygen. Therefore, increased 2,3-DPG levels triggered by tissue hypoxia will shift the hemoglobin oxygen dissociation curve to the right, increasing oxygen delivery to the tissues.

Maintenance of the RBC membrane system and hemoglobin function is dependent on energy generation through RBC metabolic pathways. There are four major RBC metabolic pathways: the Embden-Mayerhof pathway, in which most RBC adenosine triphosphate (ATP) is generated through the anaerobic breakdown of glucose; the hexose monophosphate shunt, which produces NADPH to protect RBCs from oxidative injury; the Rapoport-Luebering shunt, responsible for the production of 2,3-DPG for the control of hemoglobin oxygen affinity; and finally, the methemoglobin reduction pathway, which reduces ferric heme iron to the ferrous form to prevent hemoglobin denaturation. All four metabolic pathways are critical to RBC function.

Defects associated with any of the above described elements of RBC structure or function are related to the development and pathogenesis of the many forms of inherited and acquired RBC abnormalities that result in increased RBC destruction through intra- or extravascular hemolysis and therefore an in vivo survival of less than the normal 120 days. During a normal life span, circulating RBCs undergo metabolic and physical changes associated with the process of senescence, such as membrane vesiculation, decreased cell size, increased cell density, cytoskeletal alterations, enzymatic desialylation, and PS exposure. At the end of their life span, RBCs are recognized and removed by the macrophages in the reticuloendothelial system (RES). It has been estimated that 5 million RBCs are endocytosed by RES macrophages per second each day [202]. These RBCs are replaced by RBC reticulocytes which are released daily from the bone marrow storage pool.

Because hemoglobin and its constituent iron are highly reactive molecules that can be toxic to cells and tissues, the body has a number of protective mechanisms to avoid their accumulation in circulation. Excessive intravascular damage to red cells or transfusion of fragile or nonfunctional RBCs can result in the release of large amounts of hemoglobin into the plasma. Haptoglobin can rapidly bind free hemoglobin in circulation and clear it through the reticuloendothelial macrophages; however excessive hemolysis will exceed the haptoglobin scavenging capacity of the RES [10–13]. Conversion of excess free hemoglobin to methemoglobin is facilitated by endothelial-derived nitric oxide (NO) allowing for the rapid clearance of the oxidized hemoglobin from the circulation by the binding of hemoglobin to hemopexin and albumin [10–13]. This scavenging of the toxic free hemoglobin in circulation results in a reduced NO bioavailability, which can in turn lead to endothelial dysfunction, platelet aggregation, and oxidative injury [203–205]. Hemoglobin will accumulate within the macrophages that phagocytose senescent red cells and remove hemoglobin-laden haptoglobins and hemopexins from circulation. Through the action of cytoplasmic heme-oxygenase-1, the macrophages convert the hemoglobin into carbon monoxide, biliverdin, and free iron [206]. While some iron bound to transferrin is released into circulation, the majority of free iron accumulates within the macrophages in the form of ferritin. Under conditions where there is excessive intra- or extravascular hemolysis, the iron-sequestering capacity of ferritin and transferrin can be overwhelmed, resulting in the accumulation of free iron within the macrophages and in the circulation [207]. As free iron is highly reactive and able to generate hydroxyl radicals from hydrogen peroxide it can cause significant oxidative injury to the macrophages and tissues [208–210].

While the ability to collect, process, and store RBCs for extended periods of time has facilitated the widespread adoption of RCC transfusion as a clinical therapy, the storage of RBCs outside of the body (ex vivo) in an artificial environment impairs many natural processes required for cell viability and function. The “storage lesion” is the name given to all of the progressive changes that occur to blood components during conventional blood bank storage. Refrigerated storage of RBCs is based on the principle that biochemical events and molecular reactions can be suppressed by a reduction in temperature. Although refrigerated storage minimizes RBC injury, cellular metabolism is not completely suppressed at hypothermic temperatures and the residual metabolic activity eventually results in nutrient depletion and accumulation of cell wastes [211, 212]. The progressive biochemical and biomechanical effects of the storage lesion have been well documented [211, 213–216].

A major element of the storage lesion that has been gaining attention recently is RBC membrane injury [215–219]. Indeed, strong evidence in the current literature suggests that RBC viability—defined as posttransfusion survival of RBCs—is closely related to the structural and metabolic status of the poststorage RBC membrane [215–219]. The membrane components implicated in the hypothermic storage lesion include (i) lipid loss through microvesiculation, which leads to decreased critical hemolytic volume, increased internal RBC viscosity, and progressive spheroechinocytosis; (ii) altered RBC rheological properties such as deformability, mechanical stability, and adhesiveness; (iii) PS exposure on the membrane surface; and (iv) decreased expression of the CD47 antigen on the membrane surface [211, 215, 220–225]. Collectively, these changes in RBC membrane structure manifest as severe changes in the deformability of the stored RBCs, alterations that result in accelerated clearance of the cells from circulation [226–229].

The effect that the biochemical changes that occur to RBCs during storage have on the safety and efficacy of transfused RCCs remains unclear. Numerous studies have shown an association of RCC transfusion with increased length of stay in the hospital, impaired tissue oxygen use, proinflammatory and immunomodulatory effects, increased infections, multiple organ system failure, and ultimately increased morbidity and mortality (reviewed in [230–233]). Based on our current knowledge of the RBC physiology and the storage lesion one may propose a number of different biological pathways that may be responsible for these adverse events including the role of microvesicles (also known as microparticles [234]) in stored blood in the pathogenesis of thrombosis, inflammation, and responses to pathogens [235]; activation of platelets in the stored RCC product that could cause platelet-white blood cell aggregate complexes with procoagulant activity [236]; deformability changes that result in red cells becoming entrapped in the spleen [237]; decreased blood flow because of rigidity of transfused red cells; and decreased oxygen delivery as microvesicle-entrapped hemoglobin and free plasma hemoglobin serve as potent scavengers of nitric oxide once a patient is transfused [203]. While prospective clinical trials have failed to show a clear relationship between the duration of RCC storage and patient outcome [10, 238, 239], there continues to be a strong interest in understanding how the obvious physiological changes that occur to RBCs during ex vivo storage affect our perceptions of RCC quality.

### 4.2. Impact of Manufacturing Method on RCC Quality

Blood components are produced using methods that depend on the blood manufacturer's established procedures and on the desired products. Differences in production processes can exist across different jurisdictions and even within a single organization. Regulatory standards are applied, in part to address these concerns, but current standards are loose in terms of product standardization and focus instead, not unreasonably, on donor and recipient safety. As noted by Högman and Meryman [240] using current standards the hemoglobin content of a RCC unit may range between 30 and 90 g, a fact that transfusing clinicians are generally unaware of.

One element that is emerging as an important mediator of RCC quality is the manufacturing methods that are used to separate the RBCs, platelets, and plasma from whole blood. The method used to separate blood components from whole blood [241–244], the additive solutions used [216, 245], and other factors such as prestorage leukoreduction [246] have all been shown to affect the quality characteristics of transfused products. Therefore, it has been very difficult to achieve any level of global, or even national, standardization of blood products, which has confounded current clinical and laboratory-based studies aimed at examining adverse transfusion reactions [247].

By examining the characteristics of whole blood-derived and apheresis RCCs produced in Canada and the United States it has been shown that RCCs distributed for transfusion are not equivalent [248–251]. Similar studies in the US have evaluated RCCs prepared from whole blood donations using the PRP method for separation and RBCs collected through an automated apheresis process [242]. Collectively, these studies show that there are significant differences in the levels of hemolysis, potassium, cytokine and microparticle levels, oxidative stress, oxygen carrying capacity, deformability, and residual plasma, platelet, and leukocyte concentrations across the different methods used to manufacture an RCC.

Studies examining the link between blood component manufacturing and adverse clinical outcomes are extremely limited. This is due to a number of factors. Firstly, multicentre, randomized clinical trials examining blood component quality do not account for component manufacturing differences amongst study sites in their study designs. Participating hospitals assume that the blood components received by the blood bank are the same as those used at other sites. Only recently has there been awareness that differences in blood component manufacturing across international sites may need to be considered in secondary analysis of data. Secondly, RCCs prepared using different methods are intermixed within the inventory of a blood center and have the same product label and product code. This makes awareness of potential differences difficult for clinicians or hemovigilance programs to consider when evaluating transfusion outcomes. Thirdly, a lack of coordination between blood manufacturers, blood banks, and clinical research groups makes the randomization of specific blood components for clinical trials difficult. Fourthly, access to products manufactured using different methods can be difficult for investigators due to institutional, regional, or national purchasing agreements and regulations. Finally, limited pre- and postmarketing studies are performed to assess the impact of new manufacturing processes on patient outcomes. For example, comparison studies between apheresis and manual methods of RCC production have looked exclusively at conventional in vitro quality control parameters or radiolabelling survival studies and rarely patient outcomes [242, 252, 253]. For these reasons, the impact of blood component manufacturing and adverse transfusion outcomes has not been adequately evaluated in well-designed prospective clinical studies.

In a recently published retrospective study, the method of whole blood processing was shown to be associated with in-hospital mortality of transfused adults [254]. Patients who received fresh RCCs (≤7 days of storage) that were prepared by a whole blood filtration, top/top manufacturing method, were associated with a higher risk of in-hospital mortality than was transfusion with mid-age RBCs (stored 8–35 days) prepared by the red cell filtration, top/bottom method. This work is significant in that it suggests that adverse transfusion outcomes might be reduced by making minor changes to blood processing methods and inventory management practices.

### 4.3. Impact of Donor Factors on RCC Quality

Donor factors have long been associated with clinical outcomes in blood transfusions, ranging from fundamental transfusion practices such as ABO/Rh blood group matching and pathogen screening to more current concerns such as the increased risk of TRALI associated with plasma products from female donors [255]. Recently it has come to light that donor factors might also influence the in vitro quality of stored red blood cell products [256–259]. Analysis of quality control data from national blood banking agencies suggests that storage-associated hemolysis in stored RCCs is dependent on donor characteristics such as sex, age, ethnicity, and heritable genetic traits [256, 260–264].

Multiple groups have reported significantly lower hemolysis in units from premenopausal female donors when compared to donations from other donor populations [256, 261, 262, 265]. A number of theories have been suggested to explain this trend, based on known physiological differences between aging male and female blood donors and their circulating RBCs [261, 266, 267]. It has been suggested that a decrease in the surface area to cell volume ratio of circulating RBCs results in an increase in the osmotic fragility of circulating RBCs in older individuals [259]. Due to the association between storage hemolysis and female menopausal status, several hypotheses attribute hemolysis to the action of sex hormones [261, 266]. Testosterone has been shown to promote erythropoiesis [268], which could explain the increased hematocrit and hemoglobin levels observed in male donors compared to female donors [269]. Alternatively, it has been suggested that monthly blood loss in premenopausal female donors results in a younger population of circulating RBCs, which are less susceptible to stress and hemolysis during storage [267]. As evidenced by this list of contrasting observations and ideas, we are far from reaching a consensus on the mechanisms behind the influence of donor factors on RBC storage.

Retrospective studies have shown that there is an association between RCC donor factors and transfusion recipient outcomes. Donor-recipient sex mismatch [270–273] and/or age [271, 273] of the blood donor has been shown to be associated with an increased risk of mortality. For example, receiving a red cell product from young (<45 years) female blood donor has been associated with up to an 8% increase in the risk of death for each unit transfused compared to receiving an RCC transfusion from a male door [271]. As this observation has not been seen in retrospective analysis of French or Scandinavian patient cohorts [274, 275], more detailed retrospective data linkage studies and prospective clinical studies are needed to help further understanding the role that donor factors have on patient outcomes.

### 4.4. Quality of Irradiated RBCs

Gamma-irradiation of red blood cell concentrates is used to prevent transfusion-associated graft versus host disease (GVHD), a rare but usually fatal complication in recipients at risk. Although there is limited evidence for GVHD after transfusion of leukodepleted RCCs containing <5 × 10^6^ leukocytes/unit, irradiation of RCC is the standard of care for immune deficient patients. Irradiation is also used for transfusions from 1st- or 2nd-degree relatives, for all intrauterine transfusions and for allogeneic stem cell recipients, even if the recipient is immunocompetent [276]. Gamma-irradiation targets and inactivates residual white blood cells that may be present in RCCs [277].

It is generally known that irradiation of RCCs causes harm to the erythrocytes, with the most prominent being biochemical changes, including lipid peroxidation and membrane damage [278, 279], reducing the function and viability of RBCs which may impact their posttransfusion recovery [280]. Irradiation exacerbates many of the classical hallmarks of the red cell storage lesion [281], causing red cell swelling, decreasing deformability [282], affecting metabolism [283], increasing the release of potassium [284, 285], and increasing hemolysis [286]. Some of these changes (e.g., increased hemolysis and potassium loss) could cause harm to certain recipients [287, 288]. For these reasons, the timing of irradiation during RCC storage and the length of the postirradiation storage period are thought to be critical to the quality of gamma-irradiated RCCs [289, 290].

### 4.5. Quality of Cryopreserved RBCs

Cryopreservation is the process of preserving the biologic structure and/or function of living systems by freezing to and storage at ultralow temperatures. As with refrigerated storage, cryopreservation uses the beneficial effect of decreased temperature to suppress molecular motion and arrest metabolic and biochemical reactions. Below  −150°C, a state of “suspended animation” can be achieved in samples where there are no cryoprotectants added as there are very few biologically significant reactions or changes to the physicochemical properties of the system that occur below this temperature [291]. In contrast to hypothermic storage (at 1–6°C), RBC physiology, including hemoglobin structure and membrane and cellular energetics, is unaffected by extended storage in the frozen state [292, 293]. Cryopreservation is the only current technology that maintains ex vivo biologic function and provides long-term product storage. It has been recently suggested that cryopreserved RCCs are as safe and effective as liquid-stored products [294] and for certain patient groups may in fact be a superior product for transfusion [295].

To take advantage of the protective effects of low temperature and to successfully store RBCs for extended periods using cryopreservation techniques, damage to the cells during freezing and thawing must be minimized. The successful cryopreservation of human red blood cells became possible after the development of techniques using glycerol as a cryoprotectant to minimize both rapid and slow cooling injury [296, 297]. Over the last century, enormous progress has been made in understanding the basic elements responsible for low temperature injury in RBCs and in the development of effective techniques to protect RBCs from this cryoinjury.

Currently, there are two methods used clinically for the cryopreservation of RCCs: low glycerol/rapid cooling [298–300] and high glycerol/slow cooling [301, 302]. Low concentrations (15–20%) of glycerol, rapid cooling (>100°C/min), storage in liquid nitrogen (−196°C), or nitrogen vapour (−165°C), with rapid thawing in a 42–45°C water bath, is less commonly used method for clinical cryopreservation of RCCs. The more common cryopreservation method found in most international blood centres is the use of a high concentration of glycerol (40%) in conjunction with slow cooling (~1°C/min), storage at <−65°C, and rapid thawing in a 37°C water bath. In each case, controlled addition and removal of glycerol are required to prevent osmotic lysis of the RBC and to minimize the recipient's exposure to the chemical cryoprotectant. Both methods meet the standards set by the regulatory agencies [223, 303] requiring the removal of the cryoprotecting agent (CPA), minimal hemolysis, and recovery of at least 80% of the original red blood cell volume following deglycerolization. Deglycerolized RBCs must be stored at 1–6°C and achieve a 75% in vivo viability, 24 hours after transfusion.

### 4.6. Quality of Washed RBCs

Washed red blood cell concentrates are recommended for large-volume transfusions to potassium-sensitive patients, particularly neonates when fresh units are not available [304], for recipients who have a history of plasma-related transfusion reactions [305], and for the prevention of transfusion-related acute lung injury [306]. Recently, the use of washed RCCs has been proposed as a means to mitigate the accumulation of proinflammatory cells and molecules in stored RCCs to reduce the bioactivity of the product [307] and may be an effective means to address RBC degradation during storage.

RCCs can be washed in the blood bank using manual centrifugation and extraction methods, semiautomated cell processors, or just prior to transfusion using cell salvage devices [304, 308–310]. Manual washing and use of open cell processors hamper widespread use of washed RBCs as the resulting washed products have a limited storage duration. Due to concerns over the potential for bacterial contamination during washing of RCCs using an open processing system and decreased RBC viability when resuspended in saline rather than additive solution in the postwash period, a 24-hour expiry is applied to these products [311, 312]. This significantly constrains the window in which units can be prepared, transported, and transfused, resulting in high levels of component discards and inefficient inventory management practices. In contrast, semiautomated cell processors using closed system washing and modern additive solutions have the potential to improve the overall utilization of washed RCCs, as postwashing storage time of RCCs can be extended and the process can be more closely controlled.

The washing of RCCs has been shown to be an effective method to remove potassium, hemoglobin, and protein- or lipid-based immunomodulatory agents [310, 313–315] and may result in reduced adverse transfusion reactions [316–318]. In cardiac and neonatal patients [304, 307] and a number of animal models [319, 320], the use of washed RCCs can decrease the dose of labile plasma iron in the product and is accompanied by a blunting of posttransfusion inflammation. However, washing may also increase potassium concentration [310] while simultaneously removing a proportion of the original unit's erythrocytes, thereby resulting in a “low dose” product that may be less effective [286, 321–324]. It has been proposed that the method used to wash RCCs has an important impact on the quality characteristics of the final product [308, 309, 325, 326]. Provision of washed products is therefore usually reserved only for patients who have already developed significant adverse transfusion reactions to unwashed products.

### 4.7. Relationship between RBC In Vitro Parameters and Posttransfusion Outcomes

Quality control programs require that a number of in vitro parameters are monitored to ensure that component manufacturing is in a state of control and that RCCs meet specific physical characteristics. In most countries, blood manufacturers will ensure that RCCs meet general requirements for hematocrit, unit volume, total hemoglobin per unit, sterility, residual leukocyte concentration, RBC recovery (typically for washed or cryopreserved units), and hemolysis. As discussed below, each of these parameters is intended to ensure that the physical characteristics of the product adhere to generally acceptable clinical specifications. However, the evidence for many of these specifications, particularly for specific patient groups requiring RCC, has not been clearly established.

Hematocrit or packed cell volume is a measurement of the ratio of the volume occupied by red blood cells to the total volume of the blood sample. Hematocrit is an important factor influencing the administration of RBC units as a high hematocrit will have a reduced flow and restricted movement through leukoreduction or microparticle filters. A low hematocrit will cause the administration of large product volumes resulting in potential circulatory overload in order to administer enough red blood cells to achieve the therapeutic effect of restoring oxygen carrying capacity of the recipient. Regulatory standards vary with each jurisdiction, but in general, most countries require the hematocrit to be ≤0.80 L/L in the majority of units tested.

The mass of hemoglobin in a unit of RCC or the concentration of hemoglobin in washed and deglycerolized RCC reflects the relative dosage of RBC transfused to patients. In order for the transfusion of a RBC unit to restore the oxygen carrying capacity of the recipient the RBC unit must contain adequate hemoglobin content. The total hemoglobin per unit metric is an indirect indication of the overall efficiency of the collection and manufacturing system. Assuming a fixed collection volume of whole blood with a physiologically normal hematocrit, the total hemoglobin per unit should fall between a set range when the manufacturing process is in control.

The residual leukocyte count is the total number of leukocytes in the RCC after leukoreduction processes such as leukocyte filtration, cell washing, cryopreservation, or pathogen reduction. Residual donor leukocytes are associated with potential adverse transfusion reactions in recipients. In an effort to avoid these adverse effects most blood systems utilize universal prestorage leukoreduction of all RCCs. The residual leukocyte concentration can be used as an indirect method of the overall efficiency of the leukoreduction process and filter performance.

Sterility is the state whereby a blood product is free from infectious microorganisms. Blood manufacturing is specifically concerned with ensuring the absence of any bacteria within the blood components. Maintaining a closed sterile system is essential to prevent bacterial contamination. During blood collection, the use of a sample diversion pouch and effective skin disinfection are used to minimize the probability of introducing bacteria into the whole blood unit. Many blood systems include random RCC sterility testing as part of their quality control program.

Unit volume is the total volume of the RCC unit following processing. The administration of high volumes of transfusion products can lead to circulatory overload. In order to avoid these adverse effects but still administer enough red blood cells to achieve the action of restoring the oxygen carrying capacity of the recipient, it is important to have consistent products with a high red blood cell mass to volume ratio (i.e., hematocrit). As physicians may prescribe a specific volume of a RBC product, the labeled volume should be an accurate representation of the volume of the product in the bag.

In the case of a RBC product, RBC recovery can be defined as the percentage of the total RBC available in a whole blood collection to the actual number appearing in an end-labeled product. RBC recovery is a relative measure of the overall efficiency of the cell washing or cryopreservation process. This measure reflects the mass of RBC that are lost during processing due to hemolysis and cells becoming trapped in tubing, leukoreduction filters, or transfer bags. Recovery is a useful measure for determining the overall efficiency of a manufacturing process.

Hemolysis is an end-point measure of the overall structural integrity of the RBC. RBC hemolysis can increase due to physical processing of the cells, temperature-dependent and storage time-dependent depletion of biochemical metabolites, and changes in extracellular storage solution characteristics (pH, osmolality, and tonicity) [327]. Hemolysis of stored red cells is a normal process and increases with storage time. Some degree of hemolysis is acceptable and expected in RCCs.

In addition to the standard quality control tests for quality, many blood systems may perform additional in vitro testing to evaluate the effect that their collection, processing, storage, and distribution systems have on the quality of the RCCs they issue to hospitals [250, 328, 329]. Gross morphological examination and scoring of RBCs, measurement of potassium leakage, analysis on an automated hematology analyzer, and measurements of osmotic fragility can be used to examine any prehemolytic physical changes to the RBCs. A saline stability test and extracellular vesicle enumeration and characterization using flow cytometry and dynamic light scattering can be used to assess the state of the RBC membrane. Functional assessment of glycolytic intermediates (ATP and 2,3-diphosphoglyceric acid), hemoglobin oxygen saturation (pO_2_ and p50), and deformability (via ektacytometry or other methods) can give an indication of the O_2_ delivering capacity of the RCC. In addition, advanced metabolomics and lipidomic tools can be used to assess subtle changes that occur to the RCC. As with many in vitro tests used in the assessment of blood components, the general relevance of these measures to the actual in vivo function of the RBCs is not well established and further understanding of the clinical relevance of these assessments is needed.

A requirement to have 75% of radiolabelled, autologous red blood cells survival for 24 h in circulation remains the current standard for the assessment of RCC quality by the FDA and many other national regulatory agencies [330]. As most damaged RBCs will be removed by circulation within 24 h, those cells that remain in circulation would be expected to be viable and functioning [331]. Efforts to develop biotin-labeling techniques and other methods to identify RBCs in circulation have been pursued as replacements to the use of radionucleii in in vivo survival testing [332]; indeed, most countries in Western Europe prohibit the administration of radiolabeled substances to healthy volunteers [333]. However, the ability to remain in circulation may not be a true measure of whether a red cell can deliver oxygen or not. This has motivated groups to look to develop tools such as near-infrared spectroscopy [334] and plethysmography [335] to noninvasively monitor tissue oxygenation and microvascular blood flow in patients receiving RCC transfusions. While still in early development, these novel tools may provide a significant improvement in the ability to assess the in vivo efficacy of stored RCCs.

As the in vivo assessment of RCC quality will not be a routine quality control procedure that can be easily performed by blood manufacturers or blood transfusion services, developing in vitro tests that accurately predict in vivo survival and function is needed. As measurements of adenosine triphosphate levels in stored blood components correlate with in vivo survival, it has been suggested that levels of ATP should be at least 2.7 *μ*mol/g Hb at the end of storage [331]. Early work has shown that changes in membrane deformability and microvesiculation correlate with in vivo survival of RCCs stored in different additive solutions [336]. While correlations can be easy to identify, understanding the pathophysiological basis for the correlation and the many factors which may affect the reliability and predictability of an in vitro biomarker of quality will be critical to the widespread adoption of the targets.

As new analytical methods are developed to evaluate the quality characteristics of stored red blood cells it is important to understand the context in which they will be used. From a blood component manufacturing perspective, having sensitive in vitro tools available to detect subtle changes in the collection, manufacturing, storage, and distribution is critical to being able to detect and respond quickly to process deviations. These measures may have no relationship or correlation with the efficacy or safety of the blood for transfusion and may only be specific to assuring manufacturing process control is maintained. Similarly, in vitro quality tests that detect changes in RCCs that may be critical to patient safety or to the efficacy of the product may be completely insensitive to specific manufacturing process changes. For these reasons, blood systems should not focus on developing expertise with one specific analytical method but need to have access to a large number of analytical methods if they are to truly understand product quality and what it means to their operation and to the patients that they serve.

## 5. Overall Conclusion

Existing determinants of blood product quality provide some information that is plausibly linked to predicting posttransfusion efficacy of their transfusion. For RCC and platelet concentrates, the evidence for most indications is stronger than that currently available for transfusable plasma. Nevertheless, controversies persist among transfusionists regarding what constitutes an appropriate transfusion and/or an appropriate dose of the blood product, in different clinical settings. Emerging tests offer potential improvements in strengthening the linkages between pretransfusion quality assessments and posttransfusion clinical efficacy and in determining which blood products are indeed of the highest quality. It must be remembered that blood products, unlike traditional pharmaceutical agents, are complex, multicomponent products. Improved characterization and quality assessment have an important role to play in furthering our understanding of how these products, and their modified forms, can best be used to benefit patients. At present there is insufficient evidence to endorse replacing current blood component quality tests with more complicated technologies, such as thrombin generation assays and viscoelastic methodologies for both platelets and plasma, or plethysmography for red cells; nevertheless, it is only through continuing to investigate emerging tests and cross-correlating them with existing measures that the field can improve. The relatively simple tests currently employed as quality markers, such as FVIII activity for plasma, pH, and platelet count for platelets, and hemoglobin-related parameters for red cells, remain of value and can be conducted in many settings with locally available instrumentation.
